# (*E*)-1-(2,4-Di­nitro­benzyl­idene)-2,2-di­phenyl­hydrazine

**DOI:** 10.1107/S1600536813014360

**Published:** 2013-06-08

**Authors:** Ruth Meléndrez-Luévano, Blanca M. Cabrera-Vivas, Marcos Flores-Alamo, Juan C. Ramirez, Pedro Conde-Sánchez

**Affiliations:** a Facultad de Ciencias Químicas, Benemérita Universidad Autónoma de Puebla 72570, Puebla, Pue., Mexico; b Facultad de Química, Universidad Nacional Autónoma de Mexico, 04510 México DF, Mexico

## Abstract

In the crystal of the title compound, C_19_H_14_N_4_O_4_, the asymmetric unit consists of two discrete mol­ecules. The C=N bonds in both mol­ecules show an *E* conformation. The dihedral angles between the C atoms of the 2,4-dinitrobenzene rings and the C=N—N planes are 13.52 (9) and 13.82 (9)° for the two mol­ecules. In the crystal, C—H⋯O hydrogen bonds, mainly between the phenyl ring and the nitro group along the *b* axis.

## Related literature

For the synthesis and related structures, see: Vicini *et al.* (2002 ▶); Rollas *et al.* (2002 ▶); Mendoza *et al.* (2012 ▶). For applications of hydrazones, see: Angell *et al.* (2006 ▶); Clulow *et al.* (2008 ▶); Motherwell & Ramsay (2007 ▶).
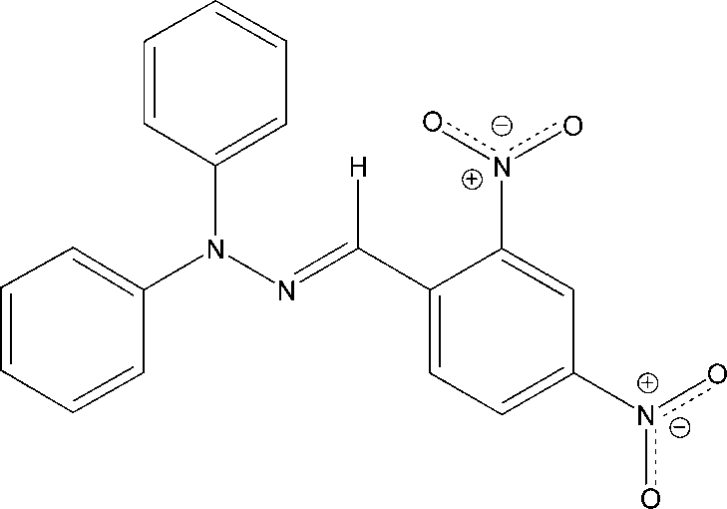


## Experimental

### 

#### Crystal data

C_19_H_14_N_4_O_4_
*M* *_r_* = 362.34
Triclinic, 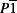

*a* = 7.0288 (6) Å
*b* = 13.5001 (7) Å
*c* = 17.9271 (11) Å
α = 91.878 (5)°
β = 93.431 (6)°
γ = 91.548 (6)°
*V* = 1696.4 (2) Å^3^
*Z* = 4
Mo *K*α radiation
μ = 0.10 mm^−1^
*T* = 298 K
0.59 × 0.38 × 0.12 mm

#### Data collection

Oxford Diffraction Xcalibur (Atlas, Gemini) diffractometer
Absorption correction: analytical (*CrysAlis PRO*; Oxford Diffraction, 2009 ▶) *T*_min_ = 0.955, *T*_max_ = 0.988
13303 measured reflections
6694 independent reflections
3465 reflections with *I* > 2σ(*I*)
*R*_int_ = 0.028

#### Refinement

*R*[*F* ^2^ > 2σ(*F* ^2^)] = 0.040
*wR*(*F* ^2^) = 0.079
*S* = 1.01
6694 reflections
487 parameters
H-atom parameters constrained
Δρ_max_ = 0.14 e Å^−3^
Δρ_min_ = −0.20 e Å^−3^

### 

Data collection: *CrysAlis CCD* (Oxford Diffraction, 2009 ▶); cell refinement: *CrysAlis RED* (Oxford Diffraction, 2009 ▶); data reduction: *CrysAlis RED*; program(s) used to solve structure: *SHELXS97* (Sheldrick, 2008 ▶); program(s) used to refine structure: *SHELXL97* (Sheldrick, 2008 ▶); molecular graphics: *ORTEP-3 for Windows* (Farrugia, 2012 ▶) and *Mercury* (Macrae *et al.*, 2006 ▶); software used to prepare material for publication: *WinGX* (Farrugia, 2012 ▶).

## Supplementary Material

Crystal structure: contains datablock(s) global, I. DOI: 10.1107/S1600536813014360/bt6909sup1.cif

Structure factors: contains datablock(s) I. DOI: 10.1107/S1600536813014360/bt6909Isup2.hkl

Supplementary material file. DOI: 10.1107/S1600536813014360/bt6909Isup3.cml

Additional supplementary materials:  crystallographic information; 3D view; checkCIF report

## Figures and Tables

**Table 1:** Hydrogen-bond geometry (Å, °)

*D*—H⋯*A*	*D*—H	H⋯*A*	*D*⋯*A*	*D*—H⋯*A*
C11*A*—H11*A*⋯O2*A* ^i^	0.93	2.59	3.306 (2)	134
C11*B*—H11*B*⋯O2*B* ^i^	0.93	2.72	3.370 (2)	128

Symmetry code: (i) 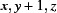.

**Fig. 1.**
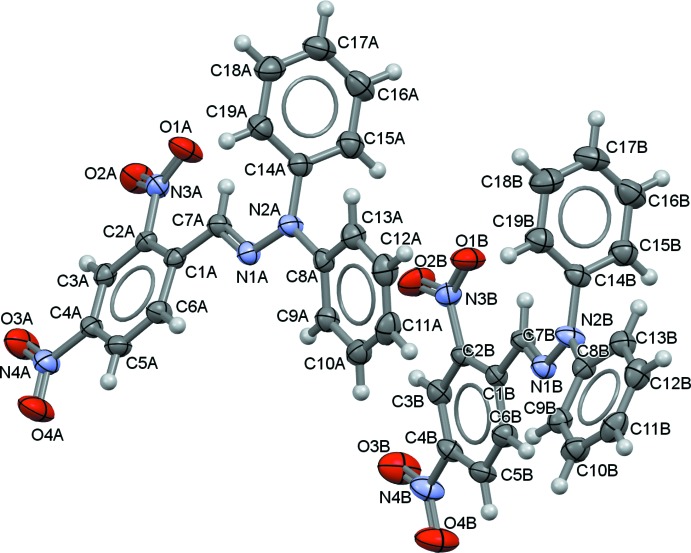
The molecular structure of the title compound. Displacement ellipsoids are drawn at the 50% probability level and H atoms are shown as circles of arbitrary size.

## supplementary crystallographic information

### Comment

Several applications of hydrazones in the industry, environmental technology, biology have been reported (Angell *et al.*, 2006). The hydrazone structure is directly related with its activity (Rollas *et al.*, 2002). Different aldehydes have been used in the condensation reaction in order to get hydrazone compounds with antibacterial and antifungal activity. It was suggested that these compounds have a better antimicrobial activity when they contain functional groups like –NO_2_ and –Cl (Vicini *et al.*, 2002).

The asymmetric unit consist of two discrete molecules of the (*E*)-1-(2,4-dinitrobenzylidene)-2,2-diphenylhydrazine I, Both *A* and *B* molecules show an *E* configuration on each of the C=N groups with diphenylhydrazine group opposite to 2,4-dinitrophenyl ring, similar to the observed in (*E*)-1-(4-nitrobenzylidene)-2,2-diphenylhydrazine (Mendoza *et al.*, 2012). The molecule *A* shows a non planar structure for a phenyl ring next to N—N group, with dihedral angles N1A—N2A—C8A—C9A and N1A—N2A—C14A—C19A of 21.3 (2) and 68.4 (2)° respectively (Table 1) analogously the non planarity structure for a phenyl ring next to N—N group in molecule *B* is observed by the N1B—N2B—C8B—C9B and N1B—N2B—C14B—C19B dihedral angles of 15.2 (2) and 96.7 (2)° respectively. The N—N average distance [1.3527 Å] is shorter than found in free diphenylhydrazine [1.418 Å] (Clulow *et al.*, 2008) and similar to related structure with 2,4 dinitrophenyl hydrazone group [1.383 (4) Å] (Motherwell & Ramsay, 2007). The dihedral angle for 2,4-dinitrophenyl rings and C=N—N planes are 12.6 (2) and 12.5 (2) ° for molecule *A* and *B* respectively. The imine bond distances C7A—N1A 1.2888 (19) Å in molecule *A* and C7B—N1B 1.293 (2) Å in molecule *B* are typical for a C=N bond.

The crystal packing (Table 1) is stabilized by intermolecular C—H···O contacts.

### Experimental

280 mg (1.53 mmol) *N*,*N*-diphenylhydrazine was dissolved in ethanol and acetic acid (0.5 ml) was added slowly into this solution while stirring. 300 mg (1.53 mmol) of 2,4-Dinitrobenzaldehyde was added drop by drop into the above solution with strong stirring and the resulting mixture was kept at atmospheric temperature until the solution became dark red transparent. After one hour and a quarter a precipitate appeared. The mixture was separated with filtration in *vacuo* and the precipitate was washed three times with cold methanol. Recrystallization was performed three times with acetonitrile, to obtain dark red crystals for X-ray analysis. Dark red crystals; yield 78%; m.p.=174–177 °C; UV λ_max_ = 443.36 nm. FT IR (film): (cm^-1^): 3119 n(C—H), 1683, 1600 n(C=N), 1513 n(Ph—NO_2_). ^1^H NMR (400 MHz, (CD_3_)_2_CO: (d/ p.p.m.): 8.73 (d, 1H, J = 2.32 Hz), 8.62 (d, 1H, J = 8.8 Hz), 8.50 (dd, 1H J= 2.32 Hz; J= 8.8 Hz), 7.68 (s, 1H), 7.55–7.51 (m, 4H), 7.35–7.27 (m, 6H). ^13^C NMR (400 MHz, (CD_3_)_2_CO: (d/ p.p.m.): 140.07, 139.05, 136.10, 135.02, 130.14, 128.78, 128.20, 126.88, 126.05, 122.49, 120.62. MS—EI: m/*z* = 362 *M*^+^. C_19_H_14_N_4_O_4_.

### Refinement

All H atoms were found in a difference map. H atoms were placed in geometrical idealized positions and were refined as riding on their parent atoms, with C—H = 0.93 Å and with *U*_iso_(H) = 1.2 *U*_eq_(C).

### Crystal data

**Table:**

C_19_H_14_N_4_O_4_	*Z* = 4
*M**_r_* = 362.34	*F*(000) = 752
Triclinic, *P*1	*D*_x_ = 1.419 Mg m^−^^3^
*a* = 7.0288 (6) Å	Mo *K*α radiation, λ = 0.71073 Å
*b* = 13.5001 (7) Å	Cell parameters from 4321 reflections
*c* = 17.9271 (11) Å	θ = 3.4–26.0°
α = 91.878 (5)°	µ = 0.10 mm^−^^1^
β = 93.431 (6)°	*T* = 298 K
γ = 91.548 (6)°	Prism, red
*V* = 1696.4 (2) Å^3^	0.59 × 0.38 × 0.12 mm

### Data collection

**Table:**

Oxford Diffraction Xcalibur (Atlas, Gemini) diffractometer	6694 independent reflections
Graphite monochromator	3465 reflections with *I* > 2σ(*I*)
Detector resolution: 10.4685 pixels mm^-1^	*R*_int_ = 0.028
ω scans	θ_max_ = 26.1°, θ_min_ = 3.4°
Absorption correction: analytical (*CrysAlis PRO*; Oxford Diffraction, 2009)	*h* = −8→7
*T*_min_ = 0.955, *T*_max_ = 0.988	*k* = −13→16
13303 measured reflections	*l* = −21→22

### Refinement

**Table:**

Refinement on *F*^2^	Primary atom site location: structure-invariant direct methods
Least-squares matrix: full	Secondary atom site location: difference Fourier map
*R*[*F*^2^ > 2σ(*F*^2^)] = 0.040	Hydrogen site location: inferred from neighbouring sites
*wR*(*F*^2^) = 0.079	H-atom parameters constrained
*S* = 1.01	*w* = 1/[σ^2^(*F*_o_^2^) + (0.0255*P*)^2^] where *P* = (*F*_o_^2^ + 2*F*_c_^2^)/3
6694 reflections	(Δ/σ)_max_ = 0.001
487 parameters	Δρ_max_ = 0.14 e Å^−^^3^
0 restraints	Δρ_min_ = −0.2 e Å^−^^3^

### Special details

**Table:**

Geometry. All s.u.'s (except the s.u. in the dihedral angle between two l.s. planes) are estimated using the full covariance matrix. The cell s.u.'s are taken into account individually in the estimation of s.u.'s in distances, angles and torsion angles; correlations between s.u.'s in cell parameters are only used when they are defined by crystal symmetry. An approximate (isotropic) treatment of cell s.u.'s is used for estimating s.u.'s involving l.s. planes.
Refinement. Refinement of *F*^2^ against ALL reflections. The weighted *R*-factor *wR* and goodness of fit *S* are based on *F*^2^, conventional *R*-factors *R* are based on *F*, with *F* set to zero for negative *F*^2^. The threshold expression of *F*^2^ > 2σ(*F*^2^) is used only for calculating *R*-factors(gt) *etc*. and is not relevant to the choice of reflections for refinement. *R*-factors based on *F*^2^ are statistically about twice as large as those based on *F*, and *R*- factors based on ALL data will be even larger.

### Fractional atomic coordinates and isotropic or equivalent isotropic displacement parameters (Å^2^)

**Table:**

	*x*	*y*	*z*	*U*_iso_*/*U*_eq_	
C1A	0.3668 (2)	−0.02195 (12)	0.81399 (8)	0.0351 (4)	
C2A	0.3795 (2)	−0.12554 (12)	0.81250 (8)	0.0351 (4)	
C3A	0.3529 (2)	−0.18098 (12)	0.87481 (8)	0.0410 (4)	
H3A	0.3639	−0.2495	0.8725	0.049*	
C4A	0.3103 (2)	−0.13346 (13)	0.93960 (8)	0.0412 (4)	
C5A	0.2958 (2)	−0.03170 (13)	0.94464 (9)	0.0475 (5)	
H5A	0.2669	−0.0001	0.9893	0.057*	
C6A	0.3250 (2)	0.02177 (13)	0.88257 (8)	0.0448 (4)	
H6A	0.3166	0.0904	0.8862	0.054*	
C7A	0.3967 (2)	0.04206 (13)	0.75145 (9)	0.0416 (4)	
H7A	0.4005	0.0166	0.7027	0.05*	
C8A	0.4850 (2)	0.29930 (12)	0.73592 (9)	0.0380 (4)	
C9A	0.5557 (2)	0.32258 (13)	0.80812 (9)	0.0443 (4)	
H9A	0.5802	0.2725	0.8414	0.053*	
C10A	0.5895 (2)	0.42039 (15)	0.83049 (10)	0.0528 (5)	
H10A	0.6363	0.4358	0.8792	0.063*	
C11A	0.5552 (3)	0.49555 (14)	0.78203 (12)	0.0597 (5)	
H11A	0.5781	0.5614	0.7977	0.072*	
C12A	0.4870 (3)	0.47210 (14)	0.71041 (11)	0.0573 (5)	
H12A	0.4641	0.5225	0.6772	0.069*	
C13A	0.4517 (2)	0.37495 (13)	0.68692 (10)	0.0493 (5)	
H13A	0.4054	0.3601	0.638	0.059*	
C14A	0.4312 (3)	0.17046 (12)	0.63452 (9)	0.0436 (4)	
C15A	0.5912 (3)	0.17379 (13)	0.59397 (10)	0.0556 (5)	
H15A	0.7088	0.1936	0.6169	0.067*	
C16A	0.5746 (3)	0.14724 (15)	0.51853 (11)	0.0667 (6)	
H16A	0.6818	0.15	0.4905	0.08*	
C17A	0.4022 (4)	0.11692 (15)	0.48480 (11)	0.0685 (6)	
H17A	0.3926	0.0983	0.4343	0.082*	
C18A	0.2445 (3)	0.11405 (15)	0.52541 (10)	0.0679 (6)	
H18A	0.1273	0.0933	0.5025	0.082*	
C19A	0.2574 (3)	0.14172 (14)	0.60044 (10)	0.0563 (5)	
H19A	0.1489	0.1409	0.6277	0.068*	
N1A	0.41773 (19)	0.13568 (10)	0.76728 (7)	0.0412 (4)	
N2A	0.4450 (2)	0.19996 (10)	0.71228 (7)	0.0460 (4)	
N3A	0.4219 (2)	−0.18328 (11)	0.74490 (8)	0.0446 (4)	
N4A	0.2742 (2)	−0.19203 (14)	1.00492 (9)	0.0588 (4)	
O1A	0.4180 (2)	−0.14424 (10)	0.68499 (7)	0.0713 (4)	
O2A	0.4573 (2)	−0.27067 (10)	0.75110 (7)	0.0690 (4)	
O3A	0.2800 (2)	−0.28198 (11)	0.99918 (7)	0.0854 (5)	
O4A	0.2412 (2)	−0.14791 (11)	1.06275 (7)	0.0868 (5)	
C1B	1.0722 (2)	0.48446 (12)	0.81436 (8)	0.0360 (4)	
C2B	1.0599 (2)	0.38024 (12)	0.81056 (8)	0.0362 (4)	
C3B	1.1091 (2)	0.32368 (13)	0.87149 (9)	0.0437 (4)	
H3B	1.0986	0.2549	0.8675	0.052*	
C4B	1.1730 (2)	0.36977 (13)	0.93732 (9)	0.0431 (4)	
C5B	1.1862 (2)	0.47202 (13)	0.94478 (9)	0.0472 (5)	
H5B	1.2284	0.5031	0.9901	0.057*	
C6B	1.1357 (2)	0.52650 (13)	0.88409 (9)	0.0462 (5)	
H6B	1.1441	0.5953	0.8895	0.055*	
C7B	1.0249 (2)	0.54984 (13)	0.75336 (9)	0.0418 (4)	
H7B	1.006	0.5257	0.7042	0.05*	
C8B	0.9320 (2)	0.80531 (12)	0.73992 (9)	0.0388 (4)	
C9B	0.8888 (2)	0.82648 (13)	0.81281 (9)	0.0457 (5)	
H9B	0.8825	0.776	0.8467	0.055*	
C10B	0.8550 (2)	0.92275 (14)	0.83500 (10)	0.0538 (5)	
H10B	0.8278	0.937	0.8843	0.065*	
C11B	0.8608 (2)	0.99803 (14)	0.78533 (11)	0.0578 (5)	
H11B	0.838	1.0628	0.8009	0.069*	
C12B	0.9002 (3)	0.97709 (14)	0.71319 (11)	0.0549 (5)	
H12B	0.903	1.0278	0.6794	0.066*	
C13B	0.9361 (2)	0.88092 (13)	0.68955 (10)	0.0482 (5)	
H13B	0.9628	0.8672	0.6401	0.058*	
C14B	0.9507 (3)	0.67717 (12)	0.63857 (9)	0.0427 (4)	
C15B	1.1080 (3)	0.66776 (14)	0.59758 (10)	0.0567 (5)	
H15B	1.2294	0.6803	0.62	0.068*	
C16B	1.0852 (3)	0.63946 (15)	0.52263 (11)	0.0677 (6)	
H16B	1.1914	0.6339	0.4944	0.081*	
C17B	0.9074 (4)	0.61979 (15)	0.49027 (11)	0.0685 (6)	
H17B	0.8927	0.6	0.44	0.082*	
C18B	0.7515 (3)	0.62890 (16)	0.53095 (11)	0.0694 (6)	
H18B	0.6305	0.6152	0.5085	0.083*	
C19B	0.7723 (3)	0.65841 (14)	0.60565 (10)	0.0562 (5)	
H19B	0.6654	0.6655	0.6334	0.067*	
N1B	1.01036 (19)	0.64297 (11)	0.77055 (7)	0.0423 (4)	
N2B	0.9722 (2)	0.70762 (10)	0.71597 (7)	0.0464 (4)	
N3B	0.99175 (19)	0.32343 (12)	0.74256 (8)	0.0462 (4)	
N4B	1.2312 (2)	0.31012 (14)	1.00096 (9)	0.0610 (5)	
O1B	0.9506 (2)	0.36593 (10)	0.68571 (7)	0.0697 (4)	
O2B	0.9778 (2)	0.23333 (10)	0.74548 (7)	0.0749 (4)	
O3B	1.2219 (2)	0.22001 (11)	0.99330 (8)	0.0905 (5)	
O4B	1.2854 (2)	0.35333 (11)	1.05901 (7)	0.0901 (5)	

### Atomic displacement parameters (Å^2^)

**Table:**

	*U*^11^	*U*^22^	*U*^33^	*U*^12^	*U*^13^	*U*^23^
C1A	0.0376 (10)	0.0321 (10)	0.0351 (9)	−0.0016 (8)	−0.0020 (7)	0.0028 (8)
C2A	0.0374 (10)	0.0326 (10)	0.0348 (9)	−0.0022 (8)	0.0020 (7)	−0.0018 (8)
C3A	0.0477 (11)	0.0318 (10)	0.0430 (10)	−0.0044 (8)	−0.0008 (8)	0.0051 (9)
C4A	0.0549 (12)	0.0377 (11)	0.0304 (9)	−0.0075 (9)	−0.0021 (8)	0.0077 (8)
C5A	0.0645 (13)	0.0428 (12)	0.0344 (10)	−0.0035 (9)	0.0010 (8)	−0.0039 (9)
C6A	0.0660 (13)	0.0305 (10)	0.0377 (10)	0.0005 (9)	0.0036 (8)	0.0008 (8)
C7A	0.0545 (12)	0.0338 (11)	0.0365 (10)	−0.0010 (8)	0.0022 (8)	0.0034 (8)
C8A	0.0429 (11)	0.0321 (10)	0.0395 (10)	0.0000 (8)	0.0043 (8)	0.0072 (9)
C9A	0.0516 (12)	0.0378 (12)	0.0439 (11)	0.0003 (9)	0.0039 (8)	0.0046 (9)
C10A	0.0499 (13)	0.0520 (13)	0.0559 (12)	−0.0037 (10)	0.0062 (9)	−0.0088 (11)
C11A	0.0595 (14)	0.0357 (12)	0.0846 (15)	−0.0021 (10)	0.0140 (11)	−0.0042 (12)
C12A	0.0649 (14)	0.0379 (13)	0.0707 (14)	0.0035 (10)	0.0090 (10)	0.0150 (11)
C13A	0.0572 (13)	0.0409 (12)	0.0499 (11)	−0.0004 (9)	0.0013 (9)	0.0089 (10)
C14A	0.0618 (13)	0.0313 (10)	0.0375 (10)	−0.0030 (9)	0.0005 (9)	0.0074 (8)
C15A	0.0662 (14)	0.0489 (13)	0.0523 (12)	−0.0005 (10)	0.0063 (10)	0.0056 (10)
C16A	0.0890 (18)	0.0579 (14)	0.0563 (14)	0.0077 (12)	0.0275 (12)	0.0038 (11)
C17A	0.111 (2)	0.0536 (14)	0.0410 (12)	−0.0023 (13)	0.0057 (13)	−0.0003 (10)
C18A	0.0920 (18)	0.0619 (15)	0.0474 (13)	−0.0137 (12)	−0.0101 (11)	0.0053 (11)
C19A	0.0671 (14)	0.0579 (14)	0.0437 (11)	−0.0080 (10)	0.0036 (9)	0.0064 (10)
N1A	0.0517 (9)	0.0342 (9)	0.0375 (8)	−0.0021 (7)	−0.0003 (6)	0.0072 (7)
N2A	0.0691 (11)	0.0348 (9)	0.0336 (8)	−0.0071 (7)	0.0006 (7)	0.0058 (7)
N3A	0.0510 (10)	0.0381 (10)	0.0448 (9)	−0.0026 (7)	0.0079 (7)	−0.0020 (8)
N4A	0.0820 (13)	0.0540 (12)	0.0399 (10)	−0.0132 (9)	0.0016 (8)	0.0081 (9)
O1A	0.1278 (13)	0.0501 (9)	0.0383 (7)	0.0053 (8)	0.0231 (7)	0.0027 (7)
O2A	0.1049 (12)	0.0346 (9)	0.0691 (9)	0.0124 (7)	0.0155 (7)	−0.0033 (7)
O3A	0.1557 (16)	0.0426 (9)	0.0587 (9)	−0.0129 (9)	0.0137 (9)	0.0140 (8)
O4A	0.1535 (15)	0.0685 (11)	0.0396 (8)	−0.0074 (10)	0.0179 (8)	0.0064 (8)
C1B	0.0402 (11)	0.0340 (11)	0.0345 (9)	0.0021 (8)	0.0068 (7)	0.0026 (8)
C2B	0.0395 (10)	0.0354 (10)	0.0339 (9)	0.0030 (8)	0.0038 (7)	−0.0022 (8)
C3B	0.0516 (12)	0.0352 (11)	0.0452 (11)	0.0066 (9)	0.0070 (8)	0.0042 (9)
C4B	0.0538 (12)	0.0420 (12)	0.0351 (10)	0.0103 (9)	0.0069 (8)	0.0083 (9)
C5B	0.0643 (13)	0.0458 (12)	0.0317 (10)	0.0033 (9)	0.0039 (8)	−0.0017 (9)
C6B	0.0635 (13)	0.0340 (11)	0.0409 (10)	0.0002 (9)	0.0032 (8)	0.0002 (9)
C7B	0.0538 (12)	0.0366 (11)	0.0349 (9)	0.0008 (8)	0.0020 (8)	0.0031 (9)
C8B	0.0406 (11)	0.0339 (11)	0.0414 (10)	0.0024 (8)	−0.0027 (7)	0.0046 (9)
C9B	0.0490 (12)	0.0419 (12)	0.0460 (11)	0.0007 (9)	0.0008 (8)	0.0024 (9)
C10B	0.0548 (13)	0.0503 (13)	0.0556 (12)	0.0033 (10)	0.0030 (9)	−0.0098 (10)
C11B	0.0521 (13)	0.0383 (12)	0.0818 (15)	0.0026 (9)	−0.0021 (10)	−0.0079 (11)
C12B	0.0593 (13)	0.0368 (12)	0.0689 (14)	0.0008 (9)	−0.0005 (10)	0.0126 (11)
C13B	0.0557 (12)	0.0423 (12)	0.0466 (11)	0.0039 (9)	0.0006 (8)	0.0067 (10)
C14B	0.0629 (13)	0.0317 (10)	0.0343 (10)	0.0052 (9)	0.0050 (9)	0.0068 (8)
C15B	0.0675 (14)	0.0511 (13)	0.0512 (12)	0.0017 (10)	0.0044 (10)	−0.0021 (10)
C16B	0.0894 (18)	0.0611 (15)	0.0548 (14)	0.0069 (12)	0.0220 (12)	−0.0021 (11)
C17B	0.109 (2)	0.0566 (14)	0.0399 (12)	0.0101 (13)	0.0019 (13)	−0.0022 (10)
C18B	0.0815 (17)	0.0714 (16)	0.0530 (13)	0.0022 (12)	−0.0137 (11)	−0.0016 (12)
C19B	0.0649 (14)	0.0565 (13)	0.0473 (12)	0.0032 (10)	0.0021 (10)	0.0032 (10)
N1B	0.0517 (9)	0.0372 (9)	0.0382 (8)	0.0025 (7)	0.0024 (6)	0.0059 (7)
N2B	0.0725 (11)	0.0360 (9)	0.0311 (8)	0.0110 (8)	0.0003 (7)	0.0069 (7)
N3B	0.0502 (10)	0.0421 (11)	0.0461 (9)	0.0024 (8)	0.0040 (7)	−0.0042 (8)
N4B	0.0870 (13)	0.0562 (12)	0.0406 (10)	0.0167 (10)	0.0008 (8)	0.0087 (9)
O1B	0.1135 (12)	0.0541 (9)	0.0390 (7)	−0.0086 (8)	−0.0118 (7)	0.0014 (7)
O2B	0.1153 (13)	0.0330 (9)	0.0734 (10)	0.0007 (8)	−0.0151 (8)	−0.0063 (7)
O3B	0.1567 (16)	0.0456 (10)	0.0692 (10)	0.0218 (10)	−0.0085 (9)	0.0147 (9)
O4B	0.1533 (16)	0.0728 (11)	0.0427 (9)	0.0195 (10)	−0.0149 (9)	0.0048 (8)

### Geometric parameters (Å, º)

**Table:**

C1A—C6A	1.397 (2)	C1B—C6B	1.399 (2)
C1A—C2A	1.403 (2)	C1B—C2B	1.406 (2)
C1A—C7A	1.459 (2)	C1B—C7B	1.456 (2)
C2A—C3A	1.3839 (19)	C2B—C3B	1.386 (2)
C2A—N3A	1.469 (2)	C2B—N3B	1.467 (2)
C3A—C4A	1.361 (2)	C3B—C4B	1.362 (2)
C3A—H3A	0.93	C3B—H3B	0.93
C4A—C5A	1.381 (2)	C4B—C5B	1.383 (2)
C4A—N4A	1.4650 (19)	C4B—N4B	1.463 (2)
C5A—C6A	1.369 (2)	C5B—C6B	1.368 (2)
C5A—H5A	0.93	C5B—H5B	0.93
C6A—H6A	0.93	C6B—H6B	0.93
C7A—N1A	1.2888 (19)	C7B—N1B	1.293 (2)
C7A—H7A	0.93	C7B—H7B	0.93
C8A—C9A	1.381 (2)	C8B—C9B	1.382 (2)
C8A—C13A	1.384 (2)	C8B—C13B	1.386 (2)
C8A—N2A	1.409 (2)	C8B—N2B	1.414 (2)
C9A—C10A	1.378 (2)	C9B—C10B	1.378 (2)
C9A—H9A	0.93	C9B—H9B	0.93
C10A—C11A	1.374 (2)	C10B—C11B	1.374 (2)
C10A—H10A	0.93	C10B—H10B	0.93
C11A—C12A	1.368 (2)	C11B—C12B	1.361 (2)
C11A—H11A	0.93	C11B—H11B	0.93
C12A—C13A	1.376 (2)	C12B—C13B	1.387 (2)
C12A—H12A	0.93	C12B—H12B	0.93
C13A—H13A	0.93	C13B—H13B	0.93
C14A—C19A	1.373 (2)	C14B—C19B	1.367 (2)
C14A—C15A	1.376 (2)	C14B—C15B	1.370 (2)
C14A—N2A	1.434 (2)	C14B—N2B	1.432 (2)
C15A—C16A	1.385 (2)	C15B—C16B	1.384 (2)
C15A—H15A	0.93	C15B—H15B	0.93
C16A—C17A	1.368 (3)	C16B—C17B	1.362 (3)
C16A—H16A	0.93	C16B—H16B	0.93
C17A—C18A	1.362 (3)	C17B—C18B	1.359 (3)
C17A—H17A	0.93	C17B—H17B	0.93
C18A—C19A	1.381 (2)	C18B—C19B	1.383 (2)
C18A—H18A	0.93	C18B—H18B	0.93
C19A—H19A	0.93	C19B—H19B	0.93
N1A—N2A	1.3534 (16)	N1B—N2B	1.3521 (16)
N3A—O1A	1.2110 (15)	N3B—O1B	1.2097 (16)
N3A—O2A	1.2195 (16)	N3B—O2B	1.2207 (17)
N4A—O4A	1.2162 (19)	N4B—O4B	1.2121 (19)
N4A—O3A	1.2173 (19)	N4B—O3B	1.2193 (19)
			
C6A—C1A—C2A	115.60 (14)	C6B—C1B—C2B	115.16 (15)
C6A—C1A—C7A	118.58 (15)	C6B—C1B—C7B	118.81 (16)
C2A—C1A—C7A	125.81 (15)	C2B—C1B—C7B	126.02 (15)
C3A—C2A—C1A	122.35 (15)	C3B—C2B—C1B	122.16 (15)
C3A—C2A—N3A	114.98 (14)	C3B—C2B—N3B	115.05 (15)
C1A—C2A—N3A	122.67 (14)	C1B—C2B—N3B	122.78 (14)
C4A—C3A—C2A	118.84 (15)	C4B—C3B—C2B	119.39 (17)
C4A—C3A—H3A	120.6	C4B—C3B—H3B	120.3
C2A—C3A—H3A	120.6	C2B—C3B—H3B	120.3
C3A—C4A—C5A	121.56 (15)	C3B—C4B—C5B	121.14 (16)
C3A—C4A—N4A	119.13 (16)	C3B—C4B—N4B	119.48 (17)
C5A—C4A—N4A	119.29 (16)	C5B—C4B—N4B	119.38 (17)
C6A—C5A—C4A	118.61 (16)	C6B—C5B—C4B	118.54 (17)
C6A—C5A—H5A	120.7	C6B—C5B—H5B	120.7
C4A—C5A—H5A	120.7	C4B—C5B—H5B	120.7
C5A—C6A—C1A	123.03 (16)	C5B—C6B—C1B	123.59 (17)
C5A—C6A—H6A	118.5	C5B—C6B—H6B	118.2
C1A—C6A—H6A	118.5	C1B—C6B—H6B	118.2
N1A—C7A—C1A	116.49 (15)	N1B—C7B—C1B	117.04 (15)
N1A—C7A—H7A	121.8	N1B—C7B—H7B	121.5
C1A—C7A—H7A	121.8	C1B—C7B—H7B	121.5
C9A—C8A—C13A	119.23 (17)	C9B—C8B—C13B	119.49 (17)
C9A—C8A—N2A	120.69 (15)	C9B—C8B—N2B	120.98 (15)
C13A—C8A—N2A	120.08 (15)	C13B—C8B—N2B	119.52 (15)
C10A—C9A—C8A	119.67 (16)	C10B—C9B—C8B	119.67 (17)
C10A—C9A—H9A	120.2	C10B—C9B—H9B	120.2
C8A—C9A—H9A	120.2	C8B—C9B—H9B	120.2
C11A—C10A—C9A	121.12 (18)	C11B—C10B—C9B	120.93 (18)
C11A—C10A—H10A	119.4	C11B—C10B—H10B	119.5
C9A—C10A—H10A	119.4	C9B—C10B—H10B	119.5
C12A—C11A—C10A	119.00 (19)	C12B—C11B—C10B	119.50 (19)
C12A—C11A—H11A	120.5	C12B—C11B—H11B	120.3
C10A—C11A—H11A	120.5	C10B—C11B—H11B	120.3
C11A—C12A—C13A	120.86 (18)	C11B—C12B—C13B	120.72 (18)
C11A—C12A—H12A	119.6	C11B—C12B—H12B	119.6
C13A—C12A—H12A	119.6	C13B—C12B—H12B	119.6
C12A—C13A—C8A	120.11 (18)	C8B—C13B—C12B	119.66 (17)
C12A—C13A—H13A	119.9	C8B—C13B—H13B	120.2
C8A—C13A—H13A	119.9	C12B—C13B—H13B	120.2
C19A—C14A—C15A	120.34 (17)	C19B—C14B—C15B	120.19 (18)
C19A—C14A—N2A	119.85 (16)	C19B—C14B—N2B	119.65 (17)
C15A—C14A—N2A	119.78 (16)	C15B—C14B—N2B	120.16 (17)
C14A—C15A—C16A	119.11 (18)	C14B—C15B—C16B	119.62 (19)
C14A—C15A—H15A	120.4	C14B—C15B—H15B	120.2
C16A—C15A—H15A	120.4	C16B—C15B—H15B	120.2
C17A—C16A—C15A	120.6 (2)	C17B—C16B—C15B	120.0 (2)
C17A—C16A—H16A	119.7	C17B—C16B—H16B	120
C15A—C16A—H16A	119.7	C15B—C16B—H16B	120
C18A—C17A—C16A	119.8 (2)	C18B—C17B—C16B	120.4 (2)
C18A—C17A—H17A	120.1	C18B—C17B—H17B	119.8
C16A—C17A—H17A	120.1	C16B—C17B—H17B	119.8
C17A—C18A—C19A	120.44 (19)	C17B—C18B—C19B	120.2 (2)
C17A—C18A—H18A	119.8	C17B—C18B—H18B	119.9
C19A—C18A—H18A	119.8	C19B—C18B—H18B	119.9
C14A—C19A—C18A	119.64 (18)	C14B—C19B—C18B	119.65 (19)
C14A—C19A—H19A	120.2	C14B—C19B—H19B	120.2
C18A—C19A—H19A	120.2	C18B—C19B—H19B	120.2
C7A—N1A—N2A	120.05 (14)	C7B—N1B—N2B	119.66 (14)
N1A—N2A—C8A	115.78 (13)	N1B—N2B—C8B	116.16 (13)
N1A—N2A—C14A	122.80 (14)	N1B—N2B—C14B	122.32 (14)
C8A—N2A—C14A	121.41 (13)	C8B—N2B—C14B	121.11 (13)
O1A—N3A—O2A	121.87 (15)	O1B—N3B—O2B	121.78 (16)
O1A—N3A—C2A	119.95 (14)	O1B—N3B—C2B	120.07 (15)
O2A—N3A—C2A	118.17 (14)	O2B—N3B—C2B	118.16 (15)
O4A—N4A—O3A	123.09 (16)	O4B—N4B—O3B	123.35 (17)
O4A—N4A—C4A	118.02 (17)	O4B—N4B—C4B	117.89 (18)
O3A—N4A—C4A	118.88 (17)	O3B—N4B—C4B	118.76 (18)
			
C6A—C1A—C2A—C3A	−0.1 (2)	C6B—C1B—C2B—C3B	−0.6 (2)
C7A—C1A—C2A—C3A	178.63 (15)	C7B—C1B—C2B—C3B	179.58 (15)
C6A—C1A—C2A—N3A	179.84 (14)	C6B—C1B—C2B—N3B	178.50 (14)
C7A—C1A—C2A—N3A	−1.4 (2)	C7B—C1B—C2B—N3B	−1.3 (2)
C1A—C2A—C3A—C4A	0.9 (2)	C1B—C2B—C3B—C4B	−0.5 (2)
N3A—C2A—C3A—C4A	−179.06 (14)	N3B—C2B—C3B—C4B	−179.67 (15)
C2A—C3A—C4A—C5A	−0.9 (2)	C2B—C3B—C4B—C5B	1.3 (3)
C2A—C3A—C4A—N4A	177.51 (14)	C2B—C3B—C4B—N4B	−177.95 (14)
C3A—C4A—C5A—C6A	0.1 (3)	C3B—C4B—C5B—C6B	−0.8 (3)
N4A—C4A—C5A—C6A	−178.28 (15)	N4B—C4B—C5B—C6B	178.37 (14)
C4A—C5A—C6A—C1A	0.7 (3)	C4B—C5B—C6B—C1B	−0.4 (3)
C2A—C1A—C6A—C5A	−0.7 (2)	C2B—C1B—C6B—C5B	1.1 (2)
C7A—C1A—C6A—C5A	−179.55 (15)	C7B—C1B—C6B—C5B	−179.12 (15)
C6A—C1A—C7A—N1A	12.6 (2)	C6B—C1B—C7B—N1B	−12.5 (2)
C2A—C1A—C7A—N1A	−166.14 (15)	C2B—C1B—C7B—N1B	167.26 (16)
C13A—C8A—C9A—C10A	0.8 (2)	C13B—C8B—C9B—C10B	−1.6 (2)
N2A—C8A—C9A—C10A	−178.48 (16)	N2B—C8B—C9B—C10B	178.39 (15)
C8A—C9A—C10A—C11A	−0.4 (3)	C8B—C9B—C10B—C11B	1.0 (3)
C9A—C10A—C11A—C12A	−0.2 (3)	C9B—C10B—C11B—C12B	0.1 (3)
C10A—C11A—C12A—C13A	0.4 (3)	C10B—C11B—C12B—C13B	−0.6 (3)
C11A—C12A—C13A—C8A	0.0 (3)	C9B—C8B—C13B—C12B	1.2 (2)
C9A—C8A—C13A—C12A	−0.6 (2)	N2B—C8B—C13B—C12B	−178.84 (15)
N2A—C8A—C13A—C12A	178.65 (16)	C11B—C12B—C13B—C8B	0.0 (3)
C19A—C14A—C15A—C16A	−0.4 (3)	C19B—C14B—C15B—C16B	0.2 (3)
N2A—C14A—C15A—C16A	−178.53 (16)	N2B—C14B—C15B—C16B	−179.22 (16)
C14A—C15A—C16A—C17A	−0.8 (3)	C14B—C15B—C16B—C17B	−0.9 (3)
C15A—C16A—C17A—C18A	0.9 (3)	C15B—C16B—C17B—C18B	0.8 (3)
C16A—C17A—C18A—C19A	0.1 (3)	C16B—C17B—C18B—C19B	0.1 (3)
C15A—C14A—C19A—C18A	1.4 (3)	C15B—C14B—C19B—C18B	0.7 (3)
N2A—C14A—C19A—C18A	179.54 (17)	N2B—C14B—C19B—C18B	−179.92 (17)
C17A—C18A—C19A—C14A	−1.3 (3)	C17B—C18B—C19B—C14B	−0.8 (3)
C1A—C7A—N1A—N2A	−179.63 (13)	C1B—C7B—N1B—N2B	178.10 (13)
C7A—N1A—N2A—C8A	−174.03 (15)	C7B—N1B—N2B—C8B	171.41 (15)
C7A—N1A—N2A—C14A	7.1 (2)	C7B—N1B—N2B—C14B	−1.3 (2)
C9A—C8A—N2A—N1A	21.3 (2)	C9B—C8B—N2B—N1B	−15.2 (2)
C13A—C8A—N2A—N1A	−157.96 (14)	C13B—C8B—N2B—N1B	164.80 (14)
C9A—C8A—N2A—C14A	−159.80 (16)	C9B—C8B—N2B—C14B	157.63 (15)
C13A—C8A—N2A—C14A	20.9 (2)	C13B—C8B—N2B—C14B	−22.4 (2)
C19A—C14A—N2A—N1A	68.4 (2)	C19B—C14B—N2B—N1B	96.7 (2)
C15A—C14A—N2A—N1A	−113.47 (18)	C15B—C14B—N2B—N1B	−83.9 (2)
C19A—C14A—N2A—C8A	−110.43 (18)	C19B—C14B—N2B—C8B	−75.7 (2)
C15A—C14A—N2A—C8A	67.7 (2)	C15B—C14B—N2B—C8B	103.7 (2)
C3A—C2A—N3A—O1A	168.42 (15)	C3B—C2B—N3B—O1B	−177.83 (14)
C1A—C2A—N3A—O1A	−11.5 (2)	C1B—C2B—N3B—O1B	3.0 (2)
C3A—C2A—N3A—O2A	−10.4 (2)	C3B—C2B—N3B—O2B	2.4 (2)
C1A—C2A—N3A—O2A	169.66 (15)	C1B—C2B—N3B—O2B	−176.76 (15)
C3A—C4A—N4A—O4A	178.13 (17)	C3B—C4B—N4B—O4B	−179.18 (17)
C5A—C4A—N4A—O4A	−3.4 (2)	C5B—C4B—N4B—O4B	1.6 (2)
C3A—C4A—N4A—O3A	−1.2 (2)	C3B—C4B—N4B—O3B	0.4 (3)
C5A—C4A—N4A—O3A	177.22 (17)	C5B—C4B—N4B—O3B	−178.81 (18)

### Hydrogen-bond geometry (Å, º)

**Table:**

*D*—H···*A*	*D*—H	H···*A*	*D*···*A*	*D*—H···*A*
C11*A*—H11*A*···O2*A*^i^	0.93	2.59	3.306 (2)	134
C11*B*—H11*B*···O2*B*^i^	0.93	2.72	3.370 (2)	128

Symmetry code: (i) *x*, *y*+1, *z*.
